# Unique Changes in Mitochondrial Genomes Associated with Reversions of S-Type Cytoplasmic Male Sterility in Maizemar

**DOI:** 10.1371/journal.pone.0023405

**Published:** 2011-08-08

**Authors:** John T. Matera, Jessica Monroe, Woodson Smelser, Susan Gabay-Laughnan, Kathleen J. Newton

**Affiliations:** 1 Division of Biological Sciences, University of Missouri, Columbia, Missouri, United States of America; 2 Department of Plant Biology, University of Illinois, Urbana, Illinois, United States of America; University of Oxford, United Kingdom

## Abstract

Cytoplasmic male sterility (CMS) in plants is usually associated with the expression of specific chimeric regions within rearranged mitochondrial genomes. Maize CMS-S plants express high amounts of a 1.6-kb mitochondrial RNA during microspore maturation, which is associated with the observed pollen abortion. This transcript carries two chimeric open reading frames, o*rf355* and *orf77,* both unique to CMS-S. CMS-S mitochondria also contain free linear DNA plasmids bearing terminal inverted repeats (TIRs). These TIRs recombine with TIR-homologous sequences that precede *orf355/orf77* within the main mitochondrial genome to produce linear ends. Transcription of the 1.6-kb RNA is initiated from a promoter within the TIRs only when they are at linear ends. Reversions of CMS-S to fertility occur in certain nuclear backgrounds and are usually associated with loss of the S plasmids and/or the sterility-associated region. We describe an unusual set of independently recovered revertants from a single maternal lineage that retain both the S plasmids and an intact *orf355/orf77* region but which do not produce the 1.6-kb RNA. A 7.3-kb inversion resulting from illegitmate recombination between 14-bp microrepeats has separated the genomic TIR sequences from the CMS-associated region. Although RNAs containing *orf355/orf77* can still be detected in the revertants, they are not highly expressed during pollen development and they are no longer initiated from the TIR promoter at a protein-stabilized linear end. They appear instead to be co-transcribed with cytochrome oxidase subunit 2. The 7.3-kb inversion was not detected in CMS-S or in other fertile revertants. Therefore, this inversion appears to be a *de novo* mutation that has continued to sort out within a single maternal lineage, giving rise to fertile progeny in successive generations.

## Introduction

Unlike the small mitochondrial genomes of animals, in which the order of the genes along the genomes tends to be conserved, the large mitochondrial genomes of seed plants usually exhibit rearrangements among populations within a single species [1]. Rearranged mitochondrial genomes are often seen in plants that exhibit maternally inherited male sterility. Rearrangements can lead to the insertion of one or more mitochondrial gene segments into different mitochondrial DNA (mtDNA) regions to create a “chimeric” region. In the best-documented cases of CMS, a novel chimeric mitochondrial gene codes for a product that is either toxic to the developing microspores or that causes premature degeneration of the tapetum (reviewed in [2]). Female fertility is usually normal.

**Figure 1:**
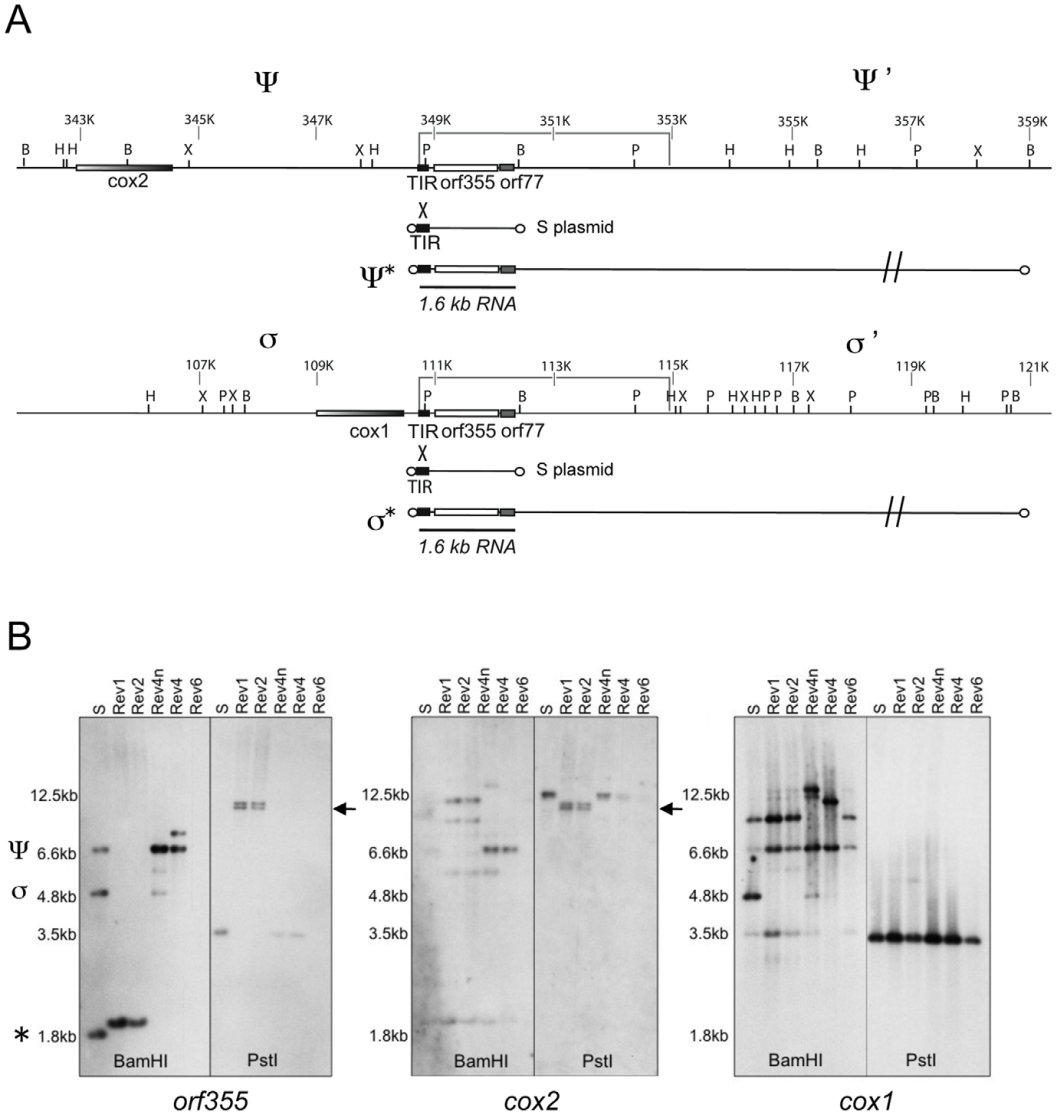
Organization of the sterility-associated regions in CMS-S mitochondria. (**A**) Origin of linear mitochondrial genomes in CMS-S mitochondria. The TIRs of protein-bound plasmids, S1 and S2, recombine with TIR sequences upstream of the chimeric region *orf355-orf77*, leading to linearization of the genome. The chimeric region is present in two copies called σ-σ' and ψ-ψ' within the “circularized” CMS-S mitochondrial genome. *Cox1* is located in σ while *cox2* is located upstream of ψ. The direction of transcription for these *cox* genes is the opposite direction from the *orf355* gene. Restriction sites for σ-σ' and ψ-ψ' are indicated: P  =  *Pst*I, H =  *Hin*dIII, B = *Bam*HI, X = *Xho*I. CMS-S genome coordinates are indicated above the lines (343K etc). The box outlines the extent of the 4.2-kb repeat. An S plasmid is represented by a short line with circles at the end (indicating protein caps). ψ* and σ* designate the linearized versions of the mitochondrial genomes after the TIR sequences in the main mtDNA have recombined with the S-plasmid TIR. The plasmid promoter within the TIR initiates transcription of the CMS-associated 1.6-kb RNA only if the chimeric region is present at a linear end [6]. (**B**) Comparisons of mtDNA from sterile (S) and revertant (Rev) CMS-S plants. Gel blots of mtDNA restriction digests were hybridized with the DIG-labeled *orf355*, *cox1* and *cox2* probes. In each panel, all the samples shown were run on the same gel, but the *Bam*HI and *Pst*I lanes were not originally adjacent (indicted by the vertical line). ψ designates the *orf355* copy near *cox2*, whereas σ designates the copy at *cox1*. The * indicates linear ends. Arrowheads point to a doublet detectable with both the *orf355* and *cox2* probes in the *Pst*I lanes. On these blots, Rev4 and Rev4n are related revertants (NCS4 striped mutant and a non-striped relative).

Most plant mitochondrial genomes map as circles [3], [4]; however, the maize CMS-S mitochondrial genome appears to exist mostly as linear molecules [5], [3], [6]. The presence of a duplicated region with adjoining chimeric open reading frames, *orf355* and *orf77*, is correlated with male sterility [7], [3]. Two free linear plasmids designated S1 and S2 are also present within CMS-S mitochondria [8]. Recombination can occur between terminal inverted repeats (TIRs) at the end of each S plasmid and TIR sequences that precede *orf355/orf77* in the main mitochondrial genome of CMS-S [5] (Figure 1A). This produces linear ends of mtDNA from which transcription of a 1.6-kb RNA initiates [6]. The male-sterile phenotype is correlated with high expression levels of the 1.6-kb mitochondrial transcript during microspore biogenesis [7], [9], [6]. The 1.6-kb RNA is present at very low levels in non-tassel tissues [6]. A constitutively expressed 2.8-kb RNA in CMS-S mitochondria also carries the *orf355/orf77* region, but this RNA is not associated with the CMS phenotype [7], [6].

There are two main ways in which the mitochondrially encoded male sterility can be overcome. One of these is through the introduction of nuclear restorer-of-fertility (*Rf*) genes [10] traditionally used by plant breeders. Alleles of these *Rf* genes encode proteins that can countermand CMS, usually by altering the sterility-associated RNA in the mitochondria. Thus, the nuclear *Rf* alleles generally do not cause mutations within the mitochondrial genome itself. For CMS-S maize, two naturally occurring restorer genes, *Rf3* and *Rf9,* have been described [11], [6].

CMS plants can also “revert” to fertility due to rearrangement mutations of the mtDNA that disrupt or alter the sterility-associated region such that it cannot be expressed. In CMS-S maize, spontaneous reversion events can be detected as fertile sectors, or totally fertile tassels, on what should be male-sterile plants (reviewed in [12]). If the sector is large or encompasses the entire tassel, it may extend into the ear as well and thus a new cytoplasmic revertant can be recovered. In addition, ears borne on plants with sterile tassels may have sectors containing predominantly the rearranged “revertant” mtDNA (ear sectors). This results in numbers of totally fertile plants in the next generation even though the parent plant bore a tassel that was male sterile. For maize CMS-S, revertant tassel sectors are observed at relatively high rates (0.1–10%) in specific nuclear backgrounds, particularly Wf9 and M825 (reviewed in [12]).

In previously reported cases, the CMS-S cytoplasmic revertant mitochondria have either lost the S plasmids or have lost part of the *orf355/orf77* region (reviewed in [12]). Here we describe several CMS-S revertants, including a set that retain both the S plasmids and an intact *orf355/orf77* region but lack the 1.6-kb CMS-associated RNA. In these cases, we show that an inversion has altered the location of the genomic copy of the TIR relative to *orf355orf/77*, so that recombination between the S plasmids and the TIR no longer produces a linear end near *orf355/orf77*.

CMS-S-like mitochondria occur naturally in some teosintes and thus predate the domestication of maize. One Mexican accession that has an S-like mitochondrial genome is designated cytoplasm 8 (Cyt8) [13], [14]. Interestingly, Cyt8 is not male sterile when introgressed into maize inbred lines that do not carry restorer-of-fertility alleles. We describe the organization of the *orf355/orf77* region in this possible CMS-S progenitor cytoplasm.

## Materials and Methods

### Plant Materials

The control CMS-S cytoplasm is carried in the B37 inbred nuclear background. Cytoplasm 8 (Cyt8) originated in a teosinte relative of maize, *Zea mays* ssp. *mexicana*, Race Central Plateau (Wilkes Collection 48703), and was bred into maize lines by recurrent backcrossing [14]. Cyt8 is similar to CMS-S in that it has S plasmids and an *orf355/orf77* region, but it is male fertile [15], [16]. This cytoplasm may be a progenitor of the sterile CMS-S cytoplasms extant in maize [13], [14].

The majority of cytoplasmic revertants in the present study (Table S1) arose in the Wf9 nuclear genotype but were found in several different “subtypes “ of CMS-S including VG, RD, and S (classified by Beckett [17]). Some subtypes of CMS-S may be identical but were collected independently and have been given different designations ([17]). Rev4 and Rev6 correspond to the “normal” relative (Rev4) and “normal” derivative (Rev6) of the previously described NCS4 (arose in RD-M825) and NCS6 (arose in RD-Wf9) abnormal growth mutants [18], [19]. The three revertants designated Rev1, Rev2, and Rev3 arose in VG -Wf9 in the same lineage and in three subsequent generations. All are descendants of the same progenitor male-sterile plant. Rev8 is a revertant that arose in RD-Wf9 and was previously studied (CR85-2053-2), [7]. Rev9 is a revertant that arose in S-Wf9 and has not been previously examined. These revertants have been maintained by crossing with pollen from the inbred Mo17 line (which does not carry any nuclear restorer alleles for CMS-S), and only fertile plants were propagated. Self-pollinations or sib-pollinations were used in cases where pollen from Mo17 was unavailable. Pedigrees of all crosses made were maintained for each revertant.

### Nucleic Acid Isolations, gel electrophoresis and DNA and RNA blot hybridizations

Mitochondrial DNA (mtDNA) was isolated from ear shoots using previously described methods [20]. Prior to lysis, the mitochondria were treated with proteinase K, except where indicated (-pK). RNA isolations were performed on mitochondrially-enriched fractions from tassels, as well as from unpollinated ear shoots. The timing of collection of tassels was important to note because the amount of the CMS-associated 1.6-kb RNA is highest in developing microspores just prior to pollen abortion [9]. “Pre-emergent” tassels were still completely surrounded by leaves and have relatively low levels of the 1.6-kb RNA. Visibly “emerging” tassels collected approximately 2 days later have higher levels of this RNA. Trizol reagent (Invitrogen #15596-018) was used for the RNA extractions.

Agarose gel electrophoresis was used to analyze nucleic acids. To test for the presence of plasmids, DNA isolated from mitochondria was directly loaded into gel lanes. Additional mtDNA samples were digested by the restriction enzymes *Bam*HI, *Hin*dIII, *Pst*I, and *Xho*1 prior to gel electrophoresis [21]. The sizes of the DNA fragments were estimated by their migrations relative to fragments of the DNA ladder (Fermentas 1-kb ladder # SM0314 and Norgen UltraRanger 1kb DNA ladder #12100). Denaturing gels were used for analysis of RNA samples (1.2% agarose and 6% formaldehyde) [22], and the sizes of the RNAs were estimated by their migrations relative to the bands of the RNA ladder (Invitrogen 0.5–10 kb RNA ladder #15623-200). Gels were stained with ethidium bromide and photographed on a UV lightbox; then the nucleic acids were blotted onto positively charged nylon membranes (GE Healthcare # RPN303B).

### Digoxigenin Probing

Digoxigenin-labeled probes for *orf355*, the mitochondrial cytochrome oxidase genes *cox1* and *cox2* and for the plasmid TIR region were generated using the DIG High Prime DNA Labeling and Detection Starter Kit II (Roche # 11-585-614-910), following the manufacturer's instructions. The gene-specific primers used to amplify probes (listed Forward 5′-3′ and Reverse 5′-3′) were: TCAGTATAGAGTCGGGGTACACTC and CAATCCACTCATCGCAGCAGGA for *orf355*; CAATGCAATAGCTTCGGTTAAGG and TCCAATTTCCGAGGACACGAACG for *cox1*; and CCATAGGCTCCTATGCTGGGAG and CTACTTGATTGCCCAACAAGGCAG for *cox2* (first exon). To generate the DIG probes, PCR reactions were performed: initial denaturation (95°C, 2 min.), 29 cycles of 95°C for 30 sec, 62°C for 30 sec, 72°C for 6 min. and a final elongation (72°C for 7 min.). Each new probe was boiled for 10 minutes and placed on ice for 1 minute before being added to a solution of DIG Easy Hyb Granules which was then hybridized to DNA or RNA gel blots. After hybridization and incubation with the anti-DIG antibody, bands were detected via a chemiluminescence reaction on X-ray film. The sizes of the nucleic acids were estimated relative to the DNA or RNA ladders included on the original gels.

### PCR Amplification and Sequencing

Primers for specific regions of the mitochondrial genome were based on the sequenced CMS-S mitochondrial genome (Genbank Accession #: DQ490951) [3], and are shown in Table 1. PCR reactions were performed using GoTaq Green Master Mix (Promega # M7122) according to the manufacturer's instructions. Following the initial denaturation (94°C, 2 min.), 29 cycles of 94°C for 30 seconds, 55°C for 30 seconds and 72°C for 4 minutes. The PCR products were analyzed by electrophoresis on 0.8% agarose gels, stained using ethidium bromide and visualized under UV light. For PCR products destined for sequencing, the samples were gel excised using the Wizard SV Gel and PCR Clean-Up System (Promega # A9282).

**Table 1:** Primer Sets for PCR.

Set	Gene Region		Primers used for PCR (5′ to 3′)	Size (bp)
1	σ-TIR	F	AGAACTATTCCAGTGAGCCCTCC	1439
		R	GGTTGGATTTATTTAGTTTGAGAC	
2	ψ-TIR	F	CATCCTCAACCAAACAAGACAGC	1366
		R	GGTTGGATTTATTTAGTTTGAGAC	
3	*orf355* to σ'	F	TCAGTATAGAGTCGGGGTACACTC	3991
		R	AGTCAAGCTGAGATCCATTACCC	
4	*orf355* to ψ'	F	TCAGTATAGAGTCGGGGTACACTC	4058
		R	TGGTCTAGTGAATGTCAGTCCG	
5	σ to σ'	F	AGAACTATTCCAGTGAGCCCTCC	5502
		R	AGTCAAGCTGAGATCCATTACCC	
6	ψ to ψ'	F	CATCCTCAACCAAACAAGACAGC	5496
		R	TGGTCTAGTGAATGTCAGTCCG	
7	Recombined ψ (ψΔ) to σ'	F	AGCCCATATCGACTTAACGGTCC	4103
		R	AGTCAAGCTGAGATCCATTACCC	
8	Recombined ψ (ψΔ) to ψ'	F	AGCCCATATCGACTTAACGGTCC	4170
		R	TGGTCTAGTGAATGTCAGTCCG	
9	Recombined ψ (ψΔ) to *orf355*	F	AGCTGAACTCGTATTCAGAGATGG	4131
		R	GTAATCTGCTTACGCAAGGGCAAC	
10	Recombined ψ (ψΔ) to *orf355*	F	CTACTTGATTGCCCAACAAGGCAG	3225
		R	GTAATCTGCTTACGCAAGGGCAAC	

Predicted PCR products are diagrammed in Figure 6.

To determine the sequences involved in the rearrangement near *orf355*, TAIL-PCR [23] was used. This method allows one to amplify a product from a known region (using a specific primer) into an unknown region (using non-specific primers). The gene-specific primer from *orf355* in reverse orientation (5′-GAGATTTGAGGCCAGCTCCCACTG-3′) was used with each of three short non-specific primers (5′-nnnnnagtc-3′, 5′-nnnnncggt-3′, 5′-nnnnnaacc-3′) for amplification under reduced stringency conditions (Table S2). Because the mitochondrial genome is so small, only the first round of amplification was needed to obtain defined PCR products. The TAIL-PCR products were excised from gels and sequenced by the University of Missouri DNA Core Facility. The rearrangement was then confirmed using two specific primers with standard PCR and sequencing techniques. The sequence information was compared to the CMS-S mitochondrial genome (Accession No. DQ490951) [3].

## Results

Spontaneous cytoplasmic reversion events are observed at a particularly high rate in the M825 nuclear background, and to a lesser extent in the Wf9 inbred nuclear background [24]. One lineage of Wf9 plants carrying the CMS-S subtype VG was unusual in that its descendants exhibited a relatively high rate of cytoplasmic reversion to fertility, with fertile plants appearing in sequential generations. The progenitor was VG-M825 crossed by Wf9 as the pollen parent five successive times; this plant was designated CMS-Orig and had a male-sterile tassel. CMS-Orig was crossed by Wf9 again and only male-sterile progeny were observed. However, it was determined that one of these sterile plants carried a sector of male fertility on the ear. A fertile plant from this revertant sector was propagated and is termed Rev1 in the current study. Male-sterile sibs of Rev1 were crossed again by Wf9 and one of them also produced a male-sterile plant with a sector of male fertility on the ear. A plant from this revertant sector was propagated and has been analyzed in the present study (Rev2). Male-sterile sibs of Rev2 were crossed by Wf9 and one of them produced three male-fertile plants out of nine, indicating a possible sector of male fertility on the ear. One of these three fertile plants was crossed by pollen from the Wf9 line, has been maintained, and is termed Rev3. Thus, Rev1, Rev2, and Rev3 arose in the same lineage in successive generations and all were recovered from ears of plants with male-sterile tassels.

Mitochondrial DNAs were isolated from plants within the CMS-VG revertant lineage (Rev1, Rev2 and Rev3), from a CMS-S revertant (Rev9), as well as from previously reported revertants from the CMS subtype RD (Rev4, Rev6 and Rev8; see Table S1). They were compared to one another and to the sequenced CMS-S mitochondrial genome (from the B37 inbred) [3]. To examine the organization of both copies of *orf355/orf77*, the purified mtDNA samples were subjected to restriction enzyme analysis (Figure 1).

Figure 1A represents the mtDNA organization surrounding the two copies of *orf355/orf77* (with restriction sites indicated on the ψ-ψ' and σ-σ' copies). Linearization of the CMS-S mitochondrial genome occurs when the small, protein-capped linear S plasmids recombine with TIR sequences within the main mitochondrial genome [5]. The TIR sequences, which are located upstream of *orf355/orf77*, lie within a pair of 4.2-kb repeats [3] (previously estimated to be 5.2 kb in length [25]). One of the repeats is near the cytochrome oxidase subunit 1 (*cox1*) gene and the other near the cytochrome oxidase subunit2 (*cox2*) gene (the σ and ψ organizations, respectively, in Figure 1 A).

DNA gel blots containing the mtDNA samples digested with restriction enzymes were hybridized with digoxigenin (DIG)-labeled probes for *orf355*, *cox1*, and *cox2* (Figure 1B). By comparing the digests in Figure 1, it can be seen that the relative positions of *orf355/orf77* and the *cox1* region in Rev1 and Rev2 are different from that of the sterile S control. In the *Bam*HI digests of the standard sterile S-genotype, three bands hybridize to the *orf355* probe: a 6.6-kb band corresponding to the *orf355* sequences near *cox2* (flanked by ψ and ψ'), a 4.8-kb band corresponding to the copy next to *cox1* (flanked by σ and σ'), and a 1.8-kb band corresponding to copies near the linear ends (Figure 1B). In contrast, Rev1 and Rev2 show a single band of approximately 2 kb. This suggests that the *orf355* region in the Rev1 and Rev2 revertants are distinctly different from the other revertants as well as the CMS-S control. This observation is supported by an examination of the hybridization pattern with *cox1* (Figure 1B). In S, the *cox1* and *orf355* probes both hybridize to the 4.8-kb *Bam*HI band (the σ region), whereas in Rev1 and Rev2, none of the *cox1*-hybridizing bands is shared with the *orf355*-hybridizing band, suggesting an altered organization of σ.

In the CMS-S mitochondrial genome, a *Pst*I site is located within the TIR sequences preceding each copy of *orf355* (Figure 1A); thus, in S-sterile plants, *orf355* does not occur on the same fragment as *cox1* or *cox2*. However, in Rev1 and Rev2, the same doublet of *Pst*I bands is detected using both the *orf355* and *cox2* probes. This result suggests that a rearrangement has occurred such that *cox2* and *orf355* are now located on the same restriction fragments in these revertants. Thus, it appears that *orf355* in Rev1 and Rev2 is not at a linear end and that it is no longer next to a TIR. To confirm this, *Bam*HI digests and DNA blot hybridizations were performed on mtDNA that was prepared in the absence of proteinase K (Figure S1). The Rev1 and Rev2 samples are different from the other revertants examined in Figure 1B. Rev6 lacks *orf355* sequences altogether. This plant is fertile because there is a deletion of the sterility-associated *orf355/orf77* regions. The two related Rev4 samples shown appear to have rearrangements affecting the *cox1* (σ) copy of *orf355/orf77.*

**Figure 2:**
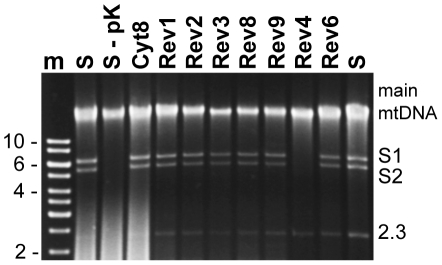
Assaying for the presence of S plasmids in sterile and revertant lines. Mitochondrial DNA after electrophoresis on a 0.8% agarose gel and staining with ethidium bromide. Revertants arising in the Wf9 nuclear background, Rev 1, 2, 3, 6, 8, and 9, have all retained S1 and S2 plasmids. Rev 4, which arose in M825, has lost S plasmids. S  =  CMS-S from B37, m  =  markers. When CMS-S mtDNA is prepared without using proteinase K (-pK), the protein-bound S plasmids, S1 and S2 do not migrate into the gel. A 2.3-kb linear plasmid unrelated to sterility is also seen.

CMS-S revertants arising in the Wf9 nuclear background retain the mitochondrial S plasmids. Gel electrophoresis of “uncut” DNA (samples not treated with restriction enzymes) confirmed the presence of the 6.4-kb S1 and the 5.4-kb S2 plasmid bands in all the samples except for Rev4 which arose in M825 (Figure 2). The linear S plasmids are stable because proteins are attached to their termini. When a proteinase K step is not included during the mtDNA isolation, the protein-bound plasmids do not migrate into the gel (Figure 2, - pK).

Nuclear background influences both the rates and types of reversion events that occur. Revertants Rev4 and Rev6 were previously described and are a normal fertile relative and a normal fertile derivative, respectively, of defective growth mutants NCS4 and NCS6 [19], [18]. Rev4 arose in the M825 nuclear background, which gives rise to fertile revertant sectors in tassels in approximately 10% of CMS-S plants [24]. Rev6 arose in the Wf9 nuclear background, which gives rise to fertile revertants at a rate of approximately 1-2% [24]. Rev4 lacks the S plasmids, which is typical of M825 revertants. All of the other revertants shown in Figure 2 (including Rev6) arose in the Wf9 nuclear background and have retained the S plasmids.

The samples labeled “S” are CMS-S mtDNA in B37, a nuclear background in which CMS-S is very stable. Cyt8 is a fertile mitochondrial genotype derived from a teosinte relative of maize that was introgressed into maize by continually crossing it with maize pollen. Cyt8 mitochondria have the S plasmids (Figure 2) and the mtDNA is quite similar to that of CMS-S [13] (see Figure S2). However, maize plants with Cyt8 mitochondria are male fertile; therefore, Cyt8 may be the progenitor cytoplasm of CMS-S [13].

**Figure 3:**
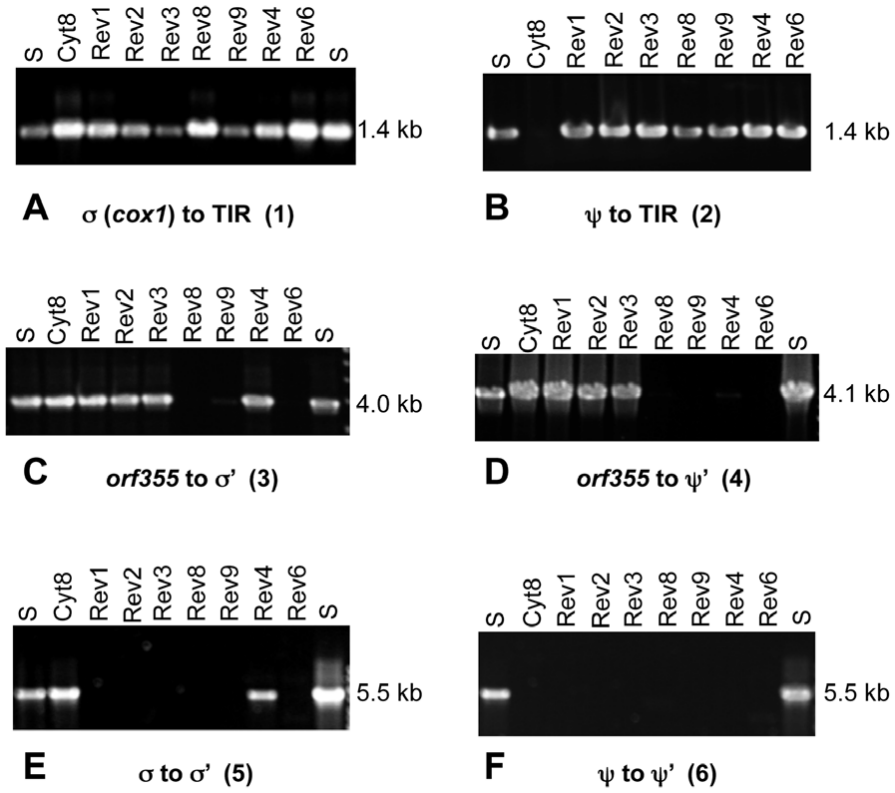
Analysis of the regions surrounding the two copies of *orf355/orf77*. PCR products amplified from ear shoot mtDNA with primer sets listed in Table 1. The predicted PCR products are diagrammed in Figure 6A. (**A**) Amplification with primer set 1, from a unique region within *cox1* (σ) to the right end of the TIR sequence. (**B**) PCR products with primers from ψ and from the right end of the TIR sequence (primer set 2, Table 1). (**C**) PCR products with primers from the beginning of *orf355* and the unique downstream σ' region (set 3). (**D**) PCR products with primers from the beginning of *orf355* and the unique downstream ψ'region (set 4). (**E**) PCR products with primers from within *cox1* (σ) and from σ' (set 5). (**F**) PCR products with primers from ψ and from ψ' (set 6).

The two copies of the *orf355/orf77* region (see Figure 1) were examined systematically, from the 5′ ends to the 3′ ends, using PCR with the primer sets shown in Table 1 (Figure 3). First we tested whether the DNA between the each of the two TIR sequences and the regions immediately upstream of them was intact (Figure 3A and 3B). The primer set for σ included a primer within *cox1* and one at the far end of the TIR (Figure 3A). Similarly the primer set for ψ included a primer near *cox2* and one at the far end of the TIR (Figure 3B). The CMS-S samples and all the revertants showed consistently good amplification. Interestingly, the ψ region between *cox2* and the TIR sequences was not amplified in the Cyt8 sample, suggesting that this region may be defective or lost in Cyt8. Restriction digest analysis of Cyt8 mtDNA and gel blot hybridization with *orf355* confirmed the loss of the ψ arrangement neighboring *orf355/orf77* (Figure S3).

The revertants were then examined for the presence of *orf355/orf77* attached to the regions downstream of the repeats. One primer was near the 5′ end of the *orf355* coding sequence and the other was designed for either σ ' or ψ '. Revertants 8, 9 and 6 showed no amplification from *orf355* into σ ' (Figure 3C). The revertants 8, 9, as well as 4 and 6 showed no (or little) amplification from *orf355* into ψ' (Figure 3D). In these cases, *orf355/orf77* itself may be partially or fully deleted (e.g. Rev6) or the downstream regions may be missing from the mitochondrial genomes. However, for some revertants, including the three samples from the same cytoplasmic revertant lineage (Rev1, Rev2, and Rev3), the *orf355/orf77* copies appear to be present, and oriented similarly to the downstream regions (Figure 3C and 3D).

Attempts to amplify upstream of the TIRs through *orf355/orf77* to downstream regions were unsuccessful for nearly all the revertants (Figure 3E and 3F). There was no amplification in any of the revertants from ψ to ψ' (Figure 3F), and only Cyt8 and Rev4 amplified from σ to σ' (Figure 3E).

To test for sequence changes within *orf355/orf77* itself, the chimeric gene was amplified using PCR from Rev1, Rev2, Rev3 and the sterile control (B37S) using primer sets 3 and 4 (Table 1). The products were gel-purified and sequenced to ensure that that no small sequence changes had occurred that could account for the recovery of fertility. In each instance, the sequences of the control and revertant samples were identical to that of the published CMS-S reference genome [3].

If there are intact *orf355/orf77* genes attached correctly to downstream regions, as well as the promoter-containing TIR sequences attached correctly to upstream regions, why are Rev1, Rev2 and Rev3 male fertile instead of male sterile? The inability to amplify across the TIR into *orf355/orf77* implicates the 127 bp that lie between the end of the TIR and the start of the *orf355* coding sequence as the region where a mutation could have occurred. As such, this could be the site of a rearrangement mutation leading to fertility.

In order to identify the sequences upstream of *orf355* in the apparent rearrangement within Rev1 mtDNA, TAIL-PCR was used [23]. This method uses a specific primer from the known region with a non-specific primer, and the PCR is conducted under varying low stringency conditions (Table S2). Only one round of amplification was sufficient to produce discrete bands from mtDNA isolated from the Rev1 mutant with the primer set: 5′-nnnnncggt-3′ and 5′GAGATTTGAGGCCAGCTCCCACTG-3′. The TAIL-PCR products were excised from gels and were sequenced by the University of Missouri's DNA Core Facility. The TAIL-PCR sequence from the largest band (Figure S4) suggested that there was an inversion event with a breakpoint between the TIR sequence and the start of *orf355*.

**Figure 4:**
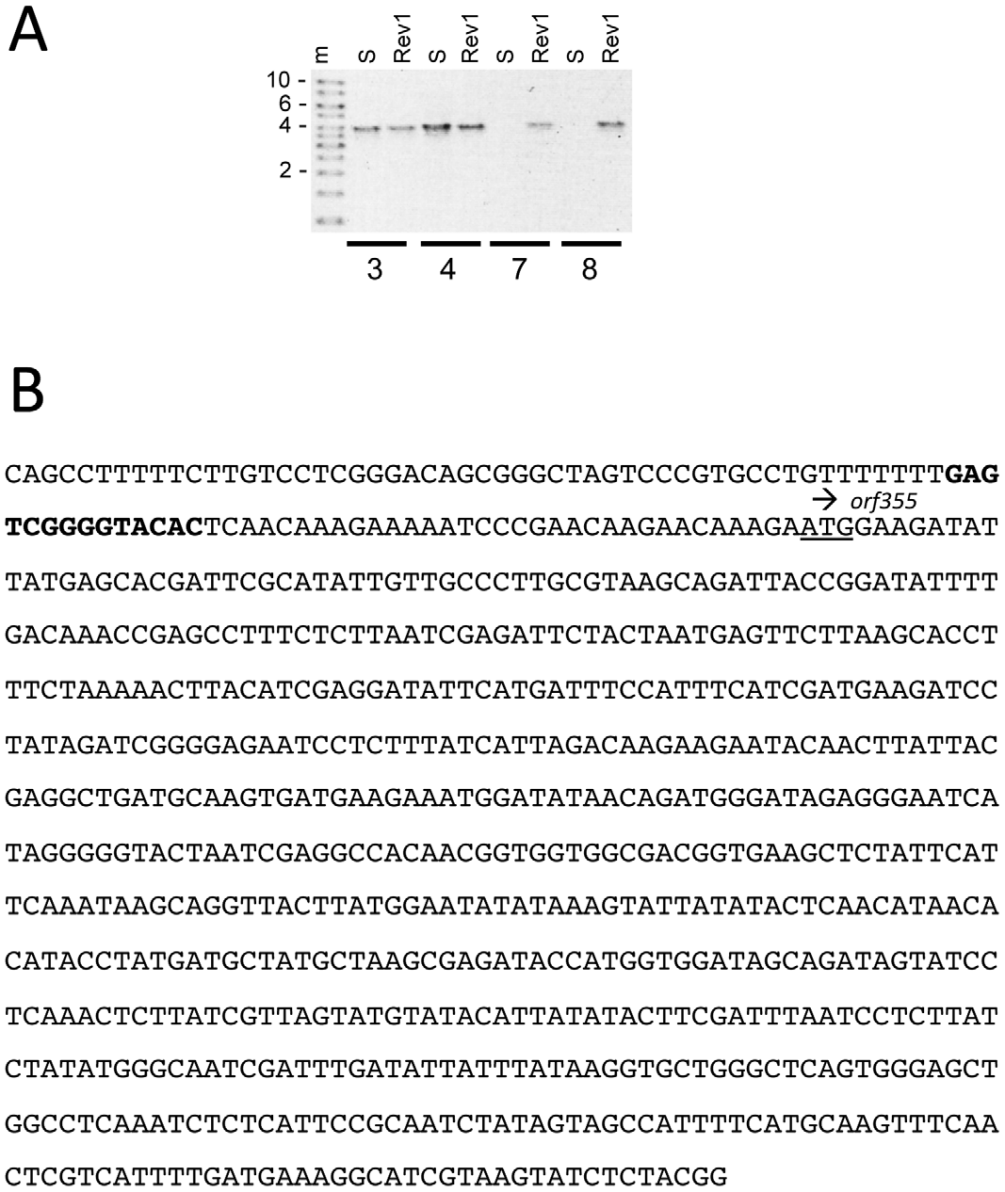
A mtDNA inversion exists in the Rev1 cytoplasmic revertant. (**A**) PCR products amplified from CMS-S (S) and Rev1 mtDNA using primer sets shown in Table 1. Set 3: amplifies from *orf355* to σ', set 4: from *orf355* to ψ', set 7: from the inverted ψ region (ψ**Δ)** to σ', set 8: from ψ**Δ** to ψ'. (**B**) Sequence data from the PCR product of primer set 8 shows a 14-bp region (bold), the probable site of recombination, located 33–47 bp before the *orf355* start codon (underlined).

**Figure 5:**
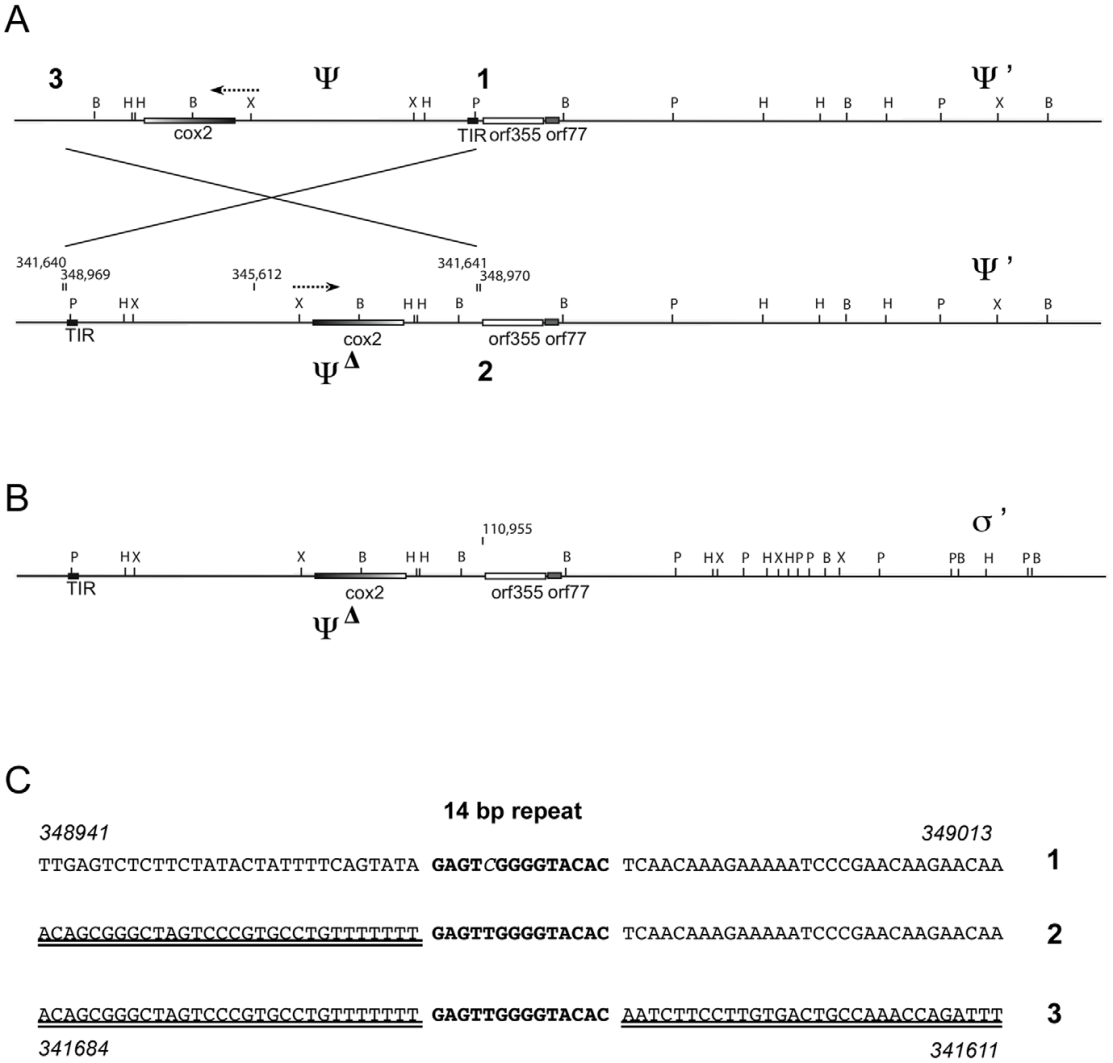
Mechanism of the mtDNA inversion. (**A**) Diagram of the inversion event in the lineage represented by Rev1, Rev2 and Rev3. A 7.3-kb inversion with breakpoints 1.2 kb after the 3′ end of *cox2* (3) and in the region between the TIR and *orf355/77* (1; 80 bp after the final nucleotide of the TIR), has separated the intact *orf355-orf77* region from its adjacent TIR sequence. Each inversion breakpoint is indicated. This inversion has placed the promoters of *cox2* in the same orientation as *orf355/orf77*, which could allow transcription of the *orf355/orf77* region from the *cox2* promoters that are now in the same orientation as *orf355/orf77* (shown by dotted arrows). (**B**) Diagram of the second copy of the ψ**Δ** inversion attached to the σ' version of *orf355/orf77*. (**C**) Examination of the sequences at the breakpoints revealed a 14-bp repeat (with 13/14 bp identity). The repeats are in opposite orientation within the CMS-S progenitor mitochondrial genotype; thus, recombination between them would lead to the inversion.

The rearrangement, designated ψ**Δ**, was then confirmed using specific primers from primer sets 7 and 8 (Table 1) with standard PCR and sequencing techniques. These primers should amplify only the inverted orientation from mtDNAs. Indeed, no band was amplified from the CMS-S control with primer sets 7 and 8, whereas discrete bands were obtained using the same primer sets with Rev1 mtDNA. (Figure 4A). The Rev1 products were sequenced (Figure 4B). The PCR results suggest that two copies of *orf355/orf77* are present in Rev1. Each is attached to ψ**Δ** at its 5′ end and to either ψ' or σ' at its 3′ end (Figure 5A and B). Further analysis of the sequences showed that the DNA directly preceding *orf355* in the revertants is found 7.3 kb upstream of *orf355* in the published CMS-S sequence (Figure 5A).

**Figure 6:**
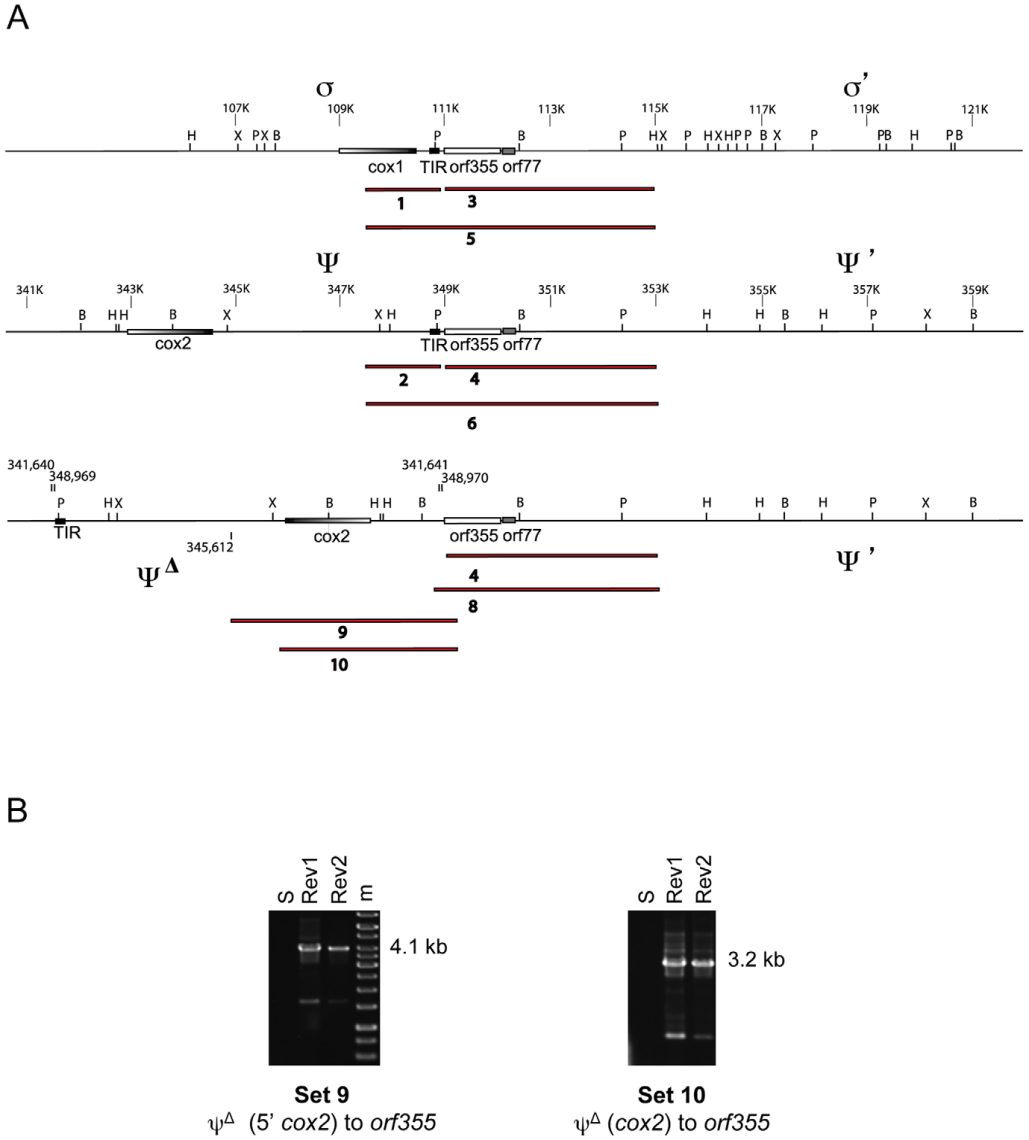
Analysis of the organization of the region upstream of orf355/orf77 in revertants with the ψΔ inversion. (**A**) Predicted PCR products for primer sets listed in Table 1. PCR products for primer sets 1–6 are shown in Figure 3. (**B**) PCR of CMS-S (S) mtDNA and two revertants (Rev1 and Rev2). Primer sets 9 and 10 amplify from *orf355* into ψ**Δ**, encompassing either the cox2 gene (Set10) or its promoter region (Set 9). Both products are specific for the inverted orientation of the *cox2* region present in the two revertants.

By comparing the rearranged sequence from the revertant to the corresponding regions in the sequenced CMS-S genome, we have been able to identify a 14-bp repeat (with 13/14 bp identity) at the inversion junction (Figure 5C). The microrepeats are in opposite orientation within the CMS-S mitochondrial genome; thus, recombination between them would result in an inversion. Short repeats have been reported to be important in generating various rearrangement and deletion mutants in plant mitochondrial DNA (reviewed in [26]). Further PCR analysis, using primer sets 9 and 10 (Table 1; Figure 6A) confirmed the reorientation of the *cox2* gene relative to *orf355/orf77* in Rev1 and Rev2 (Figure 6B). Thus, a combination of PCR and sequencing shows the existence of the ψ**Δ** inversion.

The inversion separates the TIR sequences from the *orf355/orf77* region by 7.3 kb in the revertant mitochondrial genome. Because of the inversion, the TIR promoter is reversed relative to *orf355/orf77*; therefore, transcripts that might initiate within the TIR could not include the *orf355/orf77* region. However, the *cox2* gene is now in closer proximity to *orf355/orf77* and shares the same orientation (Figure 6). Thus, in the revertant plants carrying the ψ**Δ** inversion, the *cox2* promoters are now positioned such that *cox2* and *orf355/orf77* can be co-transcribed.

**Figure 7:**
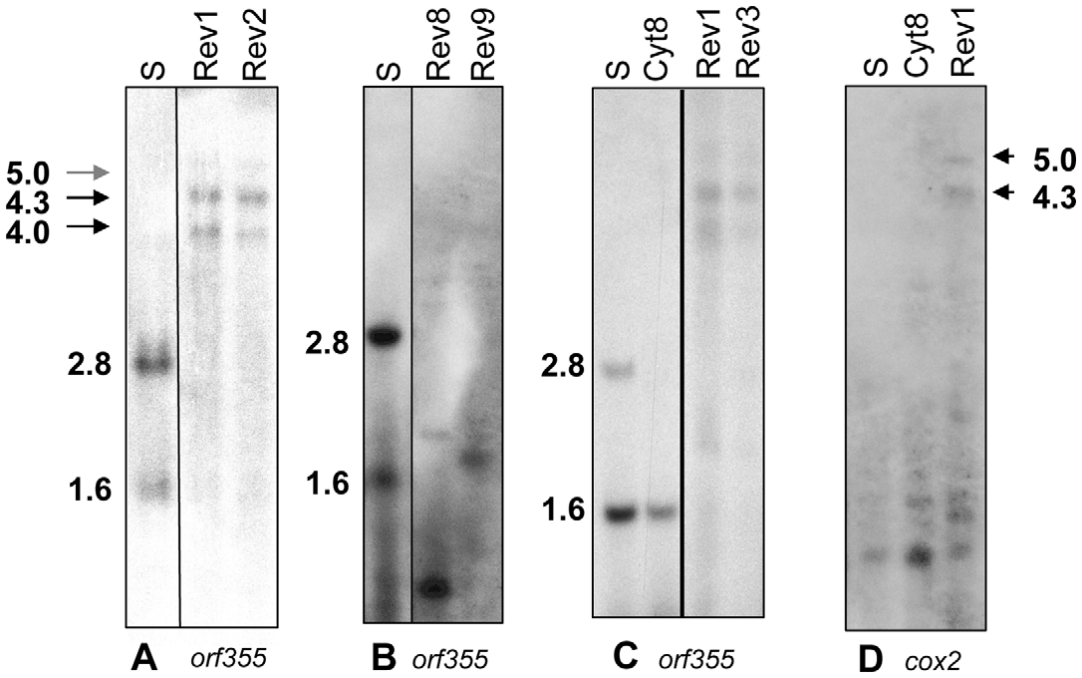
All the cytoplasmic revertants lack the sterility-associated 1.6-kb RNA. Mitochondrial RNAs from “pre-emergent” (A and B) or later “emerging” (C and D) tassels were probed with digoxigenin-labeled *orf355* and *cox2* probes. (**A**) The 1.6-kb RNA associated with sterility and the non-CMS-associated 2.8-kb RNA (originating from the ψ region) are not detectable in Rev1 and Rev2 when probed with *orf355*. A set of larger, ∼5.0, 4.3 and 4.0 kb (arrows), *orf355*-containing RNAs is detectable in the revertants. (**B**) A smaller RNA than the 1.6-kb band is detectable in Rev 8, and a slightly larger one is seen in Rev 9. (**C**) Cyt8 does have the *orf355*-hybridizing 1.6-kb RNA but it is reduced in amount compared with CMS-S. Cyt8 lacks the 2.8-kb RNA because the ψ region containing the promoter for the 2.8-kb RNA is absent from its genome. Rev3 shows the same set of larger *orf355*-containing transcripts as does Rev1. (**D**) The *cox2* probe hybridizes to a pair of larger transcripts in Rev1 that are similar in size to the two largest RNAs (∼5.0 and 4.3-kb, arrowheads) detected with *orf355*.

To test this possibility, RNA gels were run and probed with the *orf355* and *cox2* gene probes (Figure 7). In sterile (S) mitochondria, the CMS-associated 1.6-kb RNA, as well as a non-CMS-associated 2.8-kb RNA (transcribed from a promoter in the ψ region [6]), hybridizes with the *orf355* probe (Figure 7, A–C). In the revertants Rev1, Rev2, and Rev 3, neither of these RNAs is seen; instead three larger bands, with sizes of approximately 5.0 kb, 4.3 kb, and 4.0 kb hybridize with the *orf355* probe (summarized in Table S3). Similarly sized large RNAs (5.0 kb an 4.3 kb) hybridize with the *cox2* probe in these revertants (Figure 7D). This result would be expected if *orf355/orf77* were being co-transcribed from the *cox2* promoters in Rev1, Rev2 and Rev3. *Cox2* usually produces multiple RNAs because there are multiple promoters [27], [28]. The revertant lines Rev8 and Rev9, which have different rearrangements also show transcript differences when probed with *orf355* (Figure 7B).

## Discussion

The source of CMS-S mitochondria in maize is not yet known. Nuclear restorer genes for CMS-S are found within teosinte relatives of maize [15] and a phylogenetic analysis of maize mitochondrial genomes suggests that CMS-S originated in teosintes [3]. A possible fertile progenitor genotype, Cyt8, was identified in a Mexican teosinte; it carries S-like mtDNA but does not cause sterility when it is introgressed into sterility-maintainer lines of maize. We show that the teosinte-derived Cyt8 does produce the 1.6-kb RNA that is associated with S-type sterility but that the levels of this RNA are relatively low. Reduced levels of the 1.6-kb RNA have been shown previously to correlate with a loss of the sterility phenotype [6]. It was hypothesized that a “threshold” level of this RNA must be reached in the developing microspores for pollen abortion to occur. Our evidence is in accordance with this hypothesis. Cyt8 lacks the ψ copy of the *orf355/orf77*-containing repeat and produces less of the 1.6-kb RNA.

A number of cytoplasmic reversion-to-fertility events have been described previously for CMS-S in maize (reviewed in [29]). All the CMS-S revertants change the expression of the chimeric *orf355/orf77* gene such that the CMS-associated 1.6-kb transcript is no longer produced [7]. However, there is variation in the way this is accomplished.

The 1.6-kb RNA is transcribed from its promoter only when it is at a linear, protein-protected end [6]. The ability of CMS-S genomes to linearize can be eliminated when the sequences involved in the high-frequency reciprocal recombination events necessary to generate linear ends are lost; e.g., the linear S plasmids or the TIR sequences in the main mitochondrial genome. The revertants in most inbred backgrounds have indeed lost the S plasmids [30], [31], [32], [33], [7]. The Rev4 mutant studied in this paper, which arose in the M825 nuclear background, is of this type. However, revertants in the Wf9 inbred nuclear background retain the free S plasmids [34], [35], [7]. In some Wf9 revertants, including Rev6, the *orf355/orf77* region is partially or wholly deleted.

We have detailed a novel reversion event occurring in CMS-S plants in the Wf9 nuclear background in which both the chimeric *orf355/orf77* region and the S plasmids are retained and yet the plant is male fertile. The expression of the CMS-S-associated 1.6-kb RNA is no longer observed because an inversion has altered the position of the chimeric gene relative to the TIR sequences in the main mitochondrial genome. Although this novel inversion differs from any previously described CMS-S reversion event, it was found in three independent recoveries of revertant plants.

If the mutations resulting in fertility reversion occur *de novo*, we would expect independently recovered male-fertile plants to have different mitochondrial genotypes. If there is a pre-existing revertant mitochondrial sublimon [36] among the CMS-S genomes that amplifies in some plants to give fertile phenotypes, we might expect independently recovered fertile plants to have the same mutation. This has been reported for pearl millet [37], but has only been seen in maize when the revertant plants are clearly closely related; e.g. are siblings [38].

In the case of the three revertants (Rev1, Rev2 and Rev3) that share the inversion described in this study, the fertile plants are from the same maternal lineage and it is reasonable to infer that their recoveries in successive generations result from sorting out of mitochondria containing the inversion. However, this type of mtDNA change is different from those seen in the other revertants that have been analyzed in this and previous studies suggesting that it has indeed arisen *de novo*. The revertants were each recovered from ear sectors in successive generations, suggesting that the revertant genotype was at first heteroplasmic with the CMS-S genotype. During development of a heteroplasmic plant, homoplasmic cells and sectors arise from somatic segregation of the different mitochondrial genotypes [21]. Egg cells homoplasmic for mitochondria with the inversion would give rise to male-fertile plants. Heteroplasmic plants within the same lineage would continue the sorting out process and revertants would be recovered in later generations.

In maize there are at least three basic types of CMS and there are subtypes of each that correspond to different geographic isolates. These allow us to determine if the original mitochondrial genotype influences the types of rearrangement events recovered in revertant plants. We do not see any clear effect of mitochondrial genotype on the types of events recovered (reviewed in [29]). Unless plants are in the same maternal lineage, each revertant appears to be due to a different mutation.

Inbred nuclear backgrounds exist that allow us to test whether nuclear genotypes influence the rates and types of events that occur. In maize CMS-S, it is clear that the Wf9 and M825 nuclear backgrounds have high reversion rates relative to the other nuclear backgrounds examined. Nuclear genes also influence the types of events recovered. In M825, S plasmids can be lost through ectopic recombination events involving microhomologies between the S2 plasmid and different regions of the main mitochondrial genome. In Wf9, the S plasmids are retained and recombination events tend to involve the region around *orf355/orf77*, leading to deletions and rearranged segments.

Plant mitochondrial genomes are complex and actively recombine. As a consequence many rearrangements can be found among mitochondrial genomes within a species (e.g. [1], [3]). Many stable and heritable rearrangements appear to have resulted from rare recombination across short repeats, e.g. 6 bp [39], (reviewed in [26]). In our Rev1, the shared sequences at the junctions of the inversion were found to be 14 bp long. Within the sequenced CMS-S mitochondrial genome [3] these repeats are located 7.3 kb apart and exist in opposite orientation. The inversion we report is expected because recombination between inverted repeats results in an inversion of the segment between them.

## Supporting Information

Table S1 **List of cytoplasmic revertants used in this study.** Four of the revertants are described for the first time in this study. For a more complete description see plant materials in the materials and methods section.
(PDF)

Table S2 **Thermocycler program for TAIL-PCR.**
(PDF)

Table S3 **Summary of DNA configurations and transcripts containing** ***orf355.*** The open reading frame *orf355* is found either within “circularized” CMS-S mitochondrial genomes or at the linear ends resulting from recombination with the S plasmids as shown in Figure 1. In CMS revertants rev1, rev2, and rev3, *orf355* is located within the inversion shown in Figure 5, and is no longer present at linear ends. Transcripts of 2.8 kb including *orf355* can originate from within the circularized ψ region of the CMS-S genome. Because Cyt8 lacks the ψ region, the 2.8-kb RNA is not transcribed. The 1.6-kb CMS-S-associated RNA originates from linear ends immediately preceding *orf355*. In revertants rev1, rev2, and rev3, the 1.6 kb transcript is not present, but novel 5-kb and 4.3-kb RNAs including *orf355* are observed.
(PDF)

Figure S1 **TIR sequences are separated from** ***orf355*** **in Revertant 1 (Rev1).** *Bam*HI digests of (-pK) mtDNA hybridized with *orf355* (A) and TIR probes (B). The DNA was prepared without proteinase K treatment so that any *Bam*HI restriction fragments attached to proteins (e.g. protein-protected linear ends) would remain in the wells of the gel. A. The 1.8 kb *Bam*HI fragment corresponding to linear ends from CMS-S (B37S, lane 1A) is retarded in the well under these conditions, whereas the 2 kb *Bam*HI fragment in Rev1 (lane 2A) is not (Compare to Fig. 1B). Therefore, the 2-kb *Bam*HI fragment is not located at at a protein-protected linear end in the Rev1 mitochondrial genome. The *orf355* and TIR sequences are present on separate fragments in Rev1 (lane 2B) because no one fragment hybridizes to both probes. Cyt8 (Lanes 3) appears to have *orf355* only in the σ region (lacks a ψ version); it is located near TIR sequences because the σ fragment hybridizes with both probes. In contrast, Rev4 (Lane 4A) only has a ψ version of *orf355*. Rev6 (lane 5) lacks *orf355* but does have TIR sequences in its mtDNA. Bands resulting from partial digests (*p)* are indicated.
(PDF)

Figure S2 **MtDNA from Cyt8 is similar to CMS-S.** Cyt8 (8) lacks an approximately 6.3-kb *Xho*I fragment (arrow) present in CMS-S (S) mtDNA. The positions of the DNA marker bands (lambda DNA digested with *Hin*dIII) are indicated. The photo of the ethidium-bromide stained gel was inverted in Photoshop.
(PDF)

Figure S3 **Cyt8 mtDNA lacks the ψ region.** Comparison of the mtDNAs from CMS-S (S), and Cyt8 (8) samples. Upper panel: Hybridization of the DIG-labeled *orf355*-specific probe to gel blots of *Xho*I-digested mtDNA. *Xho*I sites occur outside of the repeats. When recombination occurs with a TIR of an S-plasmid (either S1 or S2), a linear end designated *σ’ or *ψ' results. The integrated σ − σ' and ψ − ψ' copies recombine with each other to give the σ −ψ' and ψ − σ' versions. Note that the bands containing ψ sequences are absent from the Cyt8 samples (arrowheads). Lower panel: Control hybridization with the *cox2* probe.
(PDF)

Figure S4 **Sequence of the Rev1 TAIL-PCR product suggested an inversion had occurred.**
(PDF)
