# Supplementary material for: Unique Changes in Mitochondrial Genomes Associated with Reversions of S-Type Cytoplasmic Male Sterility in Maizemar
Source: PLoS One. 2011 Aug 8;6(8):e23405. doi: 10.1371/journal.pone.0023405 (PMC3152571; doi:10.1371/journal.pone.0023405)
Supplement: Table S1 — List of cytoplasmic revertants used in this study. Four of the revertants are described for the first time in this study. For a more complete description see plant materials in the materials and methods section. (PDF) [file pone.0023405.s001.pdf]

Table S1. List of cytoplasmic revertants used in this study

Designation	CMS-S subtype	S1 & S2	Arose as	Cyto-Rev # (Laughnan)	Notes + reference
**Rev1**	VG	+	Total tassel<br>Possible ear sector	WF9 CR<br>87F-535-1	
**Rev2**	VG	+	Sterile tassel<br>Ear sector	WF9 CR<br>86-3634-5	
**Rev3**	VG	+	Sterile tassel<br>Ear sector	WF9 CR<br>87-4687-7	
**Rev4**	RD	-	Ear sector	M825 CR<br>82-862-7	NCS4 normal relative [19]
**Rev6**	RD	+	Tassel sector<br>Possible ear sector	WF9 CR<br>81-47-16	Same as GZ CR3 [7], NCS6 normal derivative [18]
**Rev8**	RD	+	Fertile tassel<br>Ear sector	WF9 CR<br>85-2053-2	Same as GZ CR1 [7]
**Rev9**	S	+	Ear sector	WF9 CR<br>84-1460-8	

All arose in the Wf9 nuclear background except Rev4, which arose in M825
