# Supplementary material for: Unique Changes in Mitochondrial Genomes Associated with Reversions of S-Type Cytoplasmic Male Sterility in Maizemar
Source: PLoS One. 2011 Aug 8;6(8):e23405. doi: 10.1371/journal.pone.0023405 (PMC3152571; doi:10.1371/journal.pone.0023405)
Supplement: Table S2 — Thermocycler program for TAIL-PCR. (PDF) [file pone.0023405.s002.pdf]

Table S2. Thermocycler program for TAIL-PCR

Step	Temperature	Time
1	4°C	2 min
2	92°C	3 min
3	95°C	1 min
4	94°C	30 sec
5	35°C	1 min
6	72°C	2 min
7	Repeat steps 4-6 five times	
8	94°C	30 sec
9	25°C	2 min
10	25°C +0.06°C per cycle	
11	Repeat step 10, 79 times	
12	72°C	2 min
13	94°C	30 sec
14	35°C	1 min
15	72°C	2 min
16	94°C	30 sec
17	35°C	1 min
18	72°C	2 min
19	94°C	30 sec
20	44°C	30 sec
21	72°C	2 min
22	Repeat steps 13-21, 15 times	
23	72°C	5 min
24	4°C	hold
