# Supplementary material for: Unique Changes in Mitochondrial Genomes Associated with Reversions of S-Type Cytoplasmic Male Sterility in Maizemar
Source: PLoS One. 2011 Aug 8;6(8):e23405. doi: 10.1371/journal.pone.0023405 (PMC3152571; doi:10.1371/journal.pone.0023405)
Supplement: Table S3 — Summary of DNA configurations and transcripts containing orf355. The open reading frame orf355 is found either within “circularized” CMS-S mitochondrial genomes or at the linear ends resulting from recombination with the S plasmids as shown in Figure 1. In CMS revertants rev1, rev2, and rev3, orf355 is located within the inversion shown in Figure 5, and is no longer present at linear ends. Transcripts of 2.8 kb including orf355 can originate from within the circularized ψ region of the CMS-S genome. Because Cyt8 lacks the ψ region, the 2.8-kb RNA is not transcribed. The 1.6-kb CMS-S-associated RNA originates from linear ends immediately preceding orf355. In revertants rev1, rev2, and rev3, the 1.6 kb transcript is not present, but novel 5-kb and 4.3-kb RNAs including orf355 are observed. (PDF) [file pone.0023405.s003.pdf]

Table S3. Summary of DNA configurations and transcripts containing *orf355*

Configurations of *orf355*-containing regions

	$\sigma*$	$\psi*$	$\sigma - \sigma'$	$\psi - \psi'$	$\psi\Delta - \sigma'$	$\psi\Delta - \psi'$	$\psi\Delta' - \sigma'$	$\psi\Delta' - \psi'$
CMS-S	+	+	+	+	-	-	-	-
Cyt8	+	-	-	+	-	-	-	-
Rev1	-	-	-	-	+	+	+	+
Rev2	-	-	-	-	+	+	+	+
Rev3	-	-	-	-	+	+	+	+

$\sigma*$ and $\psi*$ indicate *orf355* is near a linear end

Transcripts that include *orf355*

	1.6kb	2.8kb	5kb	4.3kb
CMS-S	+	+	-	-
Cyt8	+	-	-	-
Rev1	-	-	+	+
Rev2	-	-	+	+
Rev3	-	-	+	+
