# Supplementary figures and images for: Unique Changes in Mitochondrial Genomes Associated with Reversions of S-Type Cytoplasmic Male Sterility in Maizemar
Source: PLoS One. 2011 Aug 8;6(8):e23405. doi: 10.1371/journal.pone.0023405 (PMC3152571; doi:10.1371/journal.pone.0023405)

**Figure S1. TIR sequences are separated from *orf355* in Revertant 1 (Rev1)**

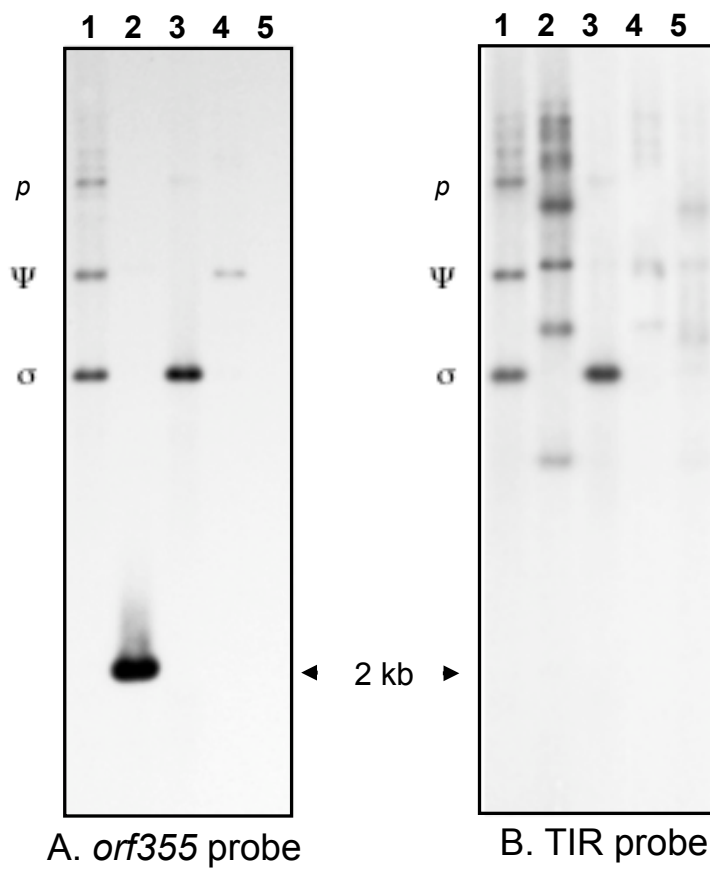

Supplement: Figure S1 — TIR sequences are separated from orf355 in Revertant 1 (Rev1). BamHI digests of (-pK) mtDNA hybridized with orf355 (A) and TIR probes (B). The DNA was prepared without proteinase K treatment so that any BamHI restriction fragments attached to proteins (e.g. protein-protected linear ends) would remain in the wells of the gel. A. The 1.8 kb BamHI fragment corresponding to linear ends from CMS-S (B37S, lane 1A) is retarded in the well under these conditions, whereas the 2 kb BamHI fragment in Rev1 (lane 2A) is not (Compare to Fig. 1B). Therefore, the 2-kb BamHI fragment is not located at at a protein-protected linear end in the Rev1 mitochondrial genome. The orf355 and TIR sequences are present on separate fragments in Rev1 (lane 2B) because no one fragment hybridizes to both probes. Cyt8 (Lanes 3) appears to have orf355 only in the σ region (lacks a ψ version); it is located near TIR sequences because the σ fragment hybridizes with both probes. In contrast, Rev4 (Lane 4A) only has a ψ version of orf355. Rev6 (lane 5) lacks orf355 but does have TIR sequences in its mtDNA. Bands resulting from partial digests (p) are indicated. (PDF) [file pone.0023405.s004.pdf]

**Figure S2. MtDNA from Cyt8 is similar to CMS-S**

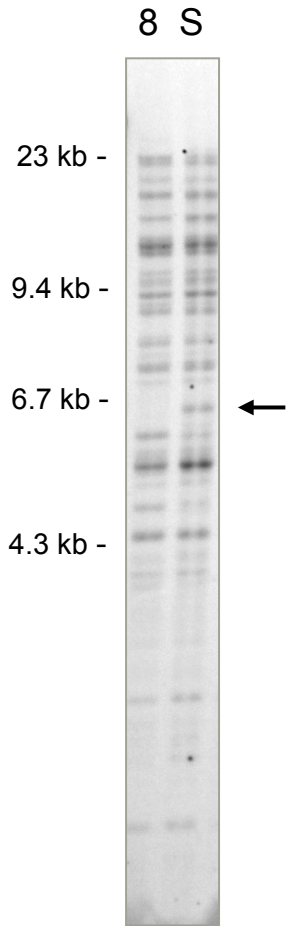

Supplement: Figure S2 — MtDNA from Cyt8 is similar to CMS-S. Cyt8 (8) lacks an approximately 6.3-kb XhoI fragment (arrow) present in CMS-S (S) mtDNA. The positions of the DNA marker bands (lambda DNA digested with HindIII) are indicated. The photo of the ethidium-bromide stained gel was inverted in Photoshop. (PDF) [file pone.0023405.s005.pdf]

**Figure S3. Cyt8 mtDNA lacks the  $\psi$  region**

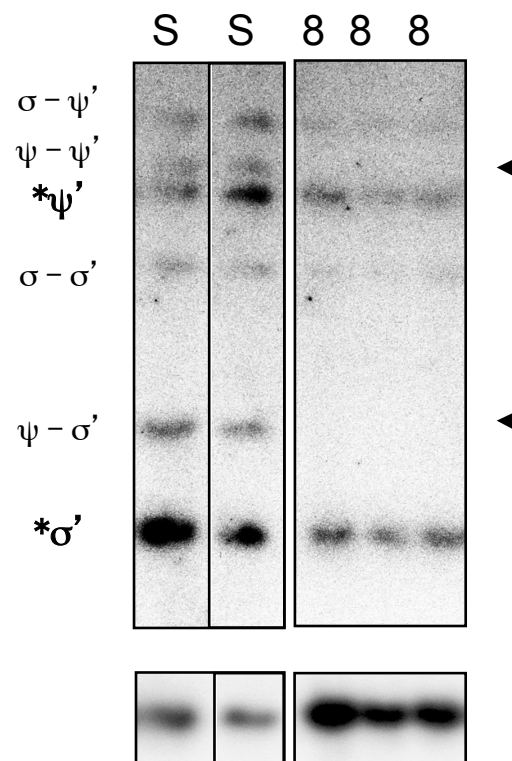

Supplement: Figure S3 — Cyt8 mtDNA lacks the ψ region. Comparison of the mtDNAs from CMS-S (S), and Cyt8 (8) samples. Upper panel: Hybridization of the DIG-labeled orf355-specific probe to gel blots of XhoI-digested mtDNA. XhoI sites occur outside of the repeats. When recombination occurs with a TIR of an S-plasmid (either S1 or S2), a linear end designated *σ’ or *ψ' results. The integrated σ − σ' and ψ − ψ' copies recombine with each other to give the σ −ψ' and ψ − σ' versions. Note that the bands containing ψ sequences are absent from the Cyt8 samples (arrowheads). Lower panel: Control hybridization with the cox2 probe. (PDF) [file pone.0023405.s006.pdf]
