# Supplementary material for: Unique Changes in Mitochondrial Genomes Associated with Reversions of S-Type Cytoplasmic Male Sterility in Maizemar
Source: PLoS One. 2011 Aug 8;6(8):e23405. doi: 10.1371/journal.pone.0023405 (PMC3152571; doi:10.1371/journal.pone.0023405)
Supplement: Figure S4 — Sequence of the Rev1 TAIL-PCR product suggested an inversion had occurred. (PDF) [file pone.0023405.s007.pdf]

Figure S4. Sequence of the Rev1 TAIL-PCR product suggested an inversion had occurred

orf355 ←

CGTGCTCATAATATCTTCCATTCTTTGTTCTTGTTCGGGATTTTTCTTTGTTGA**GTGT**

ACCCCGACTCAAAAAAACAGGCACGGGACTAGCCCGCTGTCCCGAGGACAAGAAAAAG

GCTGTGTTTAGCCTTTTCGTGCGCGATCCCAATCGTGGACCGTTAAGTCGATATGGGC

TTTATCTTAGGTTCCCCCGTATAGGTATAAAAGAAAACCCTTTTCCAGTTCATGTTAT

CGAGGACCTGGGCGATTTTGACCCGCTTCTCGGCGCTCGCCGTTTCAAGGTGAAGATC

TTCGTTAATCCTTTTTAGGATTTTTTATTTTCGTATACGCTGGCATGAGCGAATAACA

TTTTTGATTCGTTCTCGCAAGAACGAAATGGATGGTGCTTCAGGTTCAGGGTTCGGCG

GGGTGTGGCTTGTGGATGGTTCGGACGCGGCATTTTCCTCCGACTCCGATAAGTTGAG

GTACTTTTGCCAACTCTCAGAGGCGGATCCCGAAGCCAAGAATTCATCCAATTTATGA

GAGCGGATTCGCTACCGAAGAAGACCCCCAAGAGAGAAA
